# Satisfaction and compliance in hormonal contraception: the result of a multicentre clinical study on women's experience with the ethinylestradiol/norelgestromin contraceptive patch in Italy

**DOI:** 10.1186/1472-6874-9-18

**Published:** 2009-06-30

**Authors:** Pier Giorgio Crosignani, Carmine Nappi, Salvatore Ronsini, Vincenzina Bruni, Silvia Marelli, Davide Sonnino, The Italian EVRA Contrast Study Group

**Affiliations:** 1 II Institute of Obstetrics and Gynaecology, University of Milan, Milan, Italy; 2 Department of Obstetric-Gynaecological, Urological Sciences and Reproduction Medicine, University of Naples, Naples, Italy; 3 Department of Maternal and Children's Sciences, ASL SA 3 San Luca Hospital, Vallo della Lucania (SA), Italy; 4 Department of Gynaecology, Perinatology and Human Reproduction, University of Florence, Florence, Italy; 5 Medical Affairs Department, Janssen-Cilag SpA, Cologno Monzese (MI), Italy; 6 See the Acknowledgements for the complete list of members

## Abstract

**Background:**

For many women finding the right contraceptive method can be challenging and consistent and correct use over a lifetime is difficult. Even remembering to take a birth control pill every day can be a challenge. The primary objective of this study was to evaluate women's experience with a weekly ethinylestradiol/norelgestromin contraceptive patch (EE/NGMN patch), given new technologies recently developed in hormonal contraception to increase women's options in avoiding daily dosing.

**Methods:**

In 24 Italian sites, 207 women received the EE/NGMN patch for up to 6 cycles. At study end, overall satisfaction and preference, as well as compliance, efficacy and safety, were evaluated.

**Results:**

175 women (84.5%) completed the study. The overall satisfaction rate was 88%; convenience and once-a-week frequency of the patch were especially appreciated. At baseline, 82 women (39.4%) were using a contraceptive method, mainly oral contraceptives and barrier methods, but only 45.1% were very satisfied/satisfied; after 6 months with the patch, 86.3% of this subset was very satisfied/satisfied. Considering the method used in the 3 months before the study entry, 78.1% strongly preferred/preferred the patch, for convenience (53.9%), ease of use/simplicity (28.9%), fewer (9.2%) and less severe (2.6%) side effects. Compliance was very high: 1034/1110 cycles (93.2%) were completed with perfect compliance and the mean subject's compliance score was 90%. One on-therapy pregnancy occurred. The patch was safe and well tolerated: adverse events frequency was low, with predominantly single reports of each event. Most of them started and subsided during cycle 1.

**Conclusion:**

This study demonstrated that the EE/NGMN patch is associated with high satisfaction levels and excellent compliance. At study end, the majority of women indicated that they would continue using the patch.

## Background

Combined oral contraceptives are one of the most effective and commonly used forms of reversible contraception, used by more than 100 million women worldwide for family planning [1,2]. If taken exactly according to the provided instructions (perfect use), oral contraceptives are almost 100% reliable, with a failure rate of 0.1 per 100 women in the first year of use [2]; in clinical practice (typical use), the failure rate can be up to 5 per 100 women years [2], mainly due to non-compliance or imperfect compliance with the once daily regimen [3-5]. For this reason, a new technology with comparable contraceptive efficacy and safety, but not requiring daily compliance, was developed [6].

The application of transdermal technology to a birth control product was considered a milestone in transdermal drug delivery development and the ethinylestradiol/norelgestromin patch (EE/NGMN patch) was the first and unique contraceptive patch to receive worldwide regulatory approval [7]. The EE/NGMN patch marketed in Europe is a three layer 20 cm^2 ^patch containing ethinylestradiol 0.6 mg and norelgestromin 6.0 mg, applied once weekly for 3 consecutive weeks followed by a patch-free week [8]. During the 7-day wear period, the patch continuously delivers ethinylestradiol and norelgestromin, thereby avoiding peaks and troughs seen with oral contraceptives [9]. The steady-state concentrations are reached after about 48 hours. There are fewer consequences of dosing errors with the patch than with oral contraceptives: even if a scheduled patch change is delayed for 2 days during weeks 2 and 3, clinical efficacy is maintained, and backup contraception is not needed [10].

The synthetic progestin norelgestromin (17-deacetyl norgestimate, active metabolite of norgestimate) was chosen for the patch [8]. Both norgestimate and norelgestromin, virtually equivalent from metabolic and endocrine perspectives, have a low androgenicity due to their negligible binding affinities for the androgen receptor and for the sex hormone-binding globulin (SHBG) [11,12]. A clinical study showed that the patch had an effect comparable to the norgestimate-containing oral contraceptives on several key androgenic markers, suggesting this patch containing norelgestromin may improve disorders resulting from androgen excess [13]. *In vitro *studies demonstrated that norgestimate is a strong inhibitor of 5α-reductase (responsible for transforming testosterone to more potent dihydrotestosterone) in the skin [8,14]; in addition, the antiandrogenic activity of these progestins has been recently confirmed in an *in vitro *study where norgestimate and norelgestromin activity was 50% that of cyproterone acetate [15].

Several clinical studies showed that the clinical efficacy of the contraceptive patch in preventing unintended pregnancy is similar to that of oral contraceptives, with the benefit of once-weekly administration [16,17]; in a pooled analysis of three Phase III studies (3.319 women, 22.160 treatment cycles), the overall annual probability of pregnancy was 0.8% and the method failure probability was 0.6%, without differences across age [18]. The follicular size and the incidence of ovulation were significantly reduced in contraceptive patch users compared with oral contraceptive users, both in normal cycles and after planned dosing errors [19].

A pooled analysis of the same three Phase III studies previously mentioned, but limited to the subset of North America centers (1.785 women, 11.772 treatment cycles), showed that compliance (adherence to the regimen or perfect use) with the contraceptive patch was remarkably good: the rate of cycles with perfect use of the patch ranged within age groups from 88.1% to 91.0%, and adherence to the weekly schedule was significantly better than with daily oral contraceptives (p < 0.001), especially in younger women (< 20 years: 67.7% vs 87.8%) [20-22]. Another pooled analysis of the same three Phase III studies, integrated by 30 women from a three-period crossover exercise study (3.349 women, 70.639 patches worn), showed that the reliability of adhesion of the contraceptive patch was excellent and consistent across all studies [23]. Heat, humidity and exercise did not affect adhesion [23].

Finally, the adverse effect profile of the contraceptive patch is similar to that of oral contraceptives (most frequent adverse events: headache and nausea), with the exception of a higher incidence of application site reactions, breast discomfort and dysmenorrhoea in the patch group, most of them mild or moderate in severity [24]. Recent epidemiologic case-control studies conducted in the United States reported that the risk (i.e., odds ratio OR) of venous thromboembolism with the contraceptive patch compared to that of oral contraceptives containing ethinylestradiol 35 μg and norgestimate or ethinylestradiol 30 μg and levonorgestrel ranged from 0.9 (indicating no increase in risk, 95% CI 0.5–1.6) to 2.4 (indicating an approximate doubling of risk, 95% CI 1.1–5.5) [25-29].

However, in the contraceptive setting, other factors beyond efficacy and safety of the method (i.e. simplicity and ease of use, women's acceptability, satisfaction, additional benefits) can influence women's choice, adherence and persistence of use. In general, nearly half of contraception users are not completely satisfied with their current method: actual or expected side effects, difficulty of use, worry about effectiveness and reduced sexual pleasure are just some of the many reasons women give for being dissatisfied. Obviously, women who are not completely satisfied are more likely than satisfied users to make mistakes (e.g. gap in use, incorrect or inconsistent use) and to discontinue the method, putting themselves at high risk of unintended pregnancy [3]. So, contraceptive success and long-term adherence is strongly related to the subject's satisfaction [3].

In order to investigate women's experience with the EE/NGMN patch, a pan-European study, named "EVRA Contrast" (NRGEEP-CON-402), was carried out [30,31]. The primary objective of the study was to evaluate, specifically, user satisfaction and, if applicable, user preference in comparison with the previous contraceptive method. The secondary objectives were to monitor contraceptive efficacy, safety, and user compliance with the transdermal patch. Italy took part in this study, but with different timelines compared to other countries, because Italian Investigators added an additional sub-study to assess body composition with bioelectrical impedance analysis (BIA). Italian data regarding women's experience with the patch are now presented in this paper.

## Methods

### Study design and visits

In Italy, this open-label, single-arm multicentre study was conducted in 24 sites, between June 2004 and November 2005, in accordance with the principles of the International Conference on Harmonization (ICH) Good Clinical Practice (GCP) and the latest revision of the Declaration of Helsinki; ethics approval was obtained in writing by the Independent Ethics Committees (IECs) of each study centre. After informed consent signature, study participants were scheduled to receive the EE/NGMN contraceptive transdermal patch (EVRA, Janssen-Cilag International N.V., Belgium) for 24 weeks (6 cycles of 4 weeks).

A screening visit (Visit 1: eligibility, informed consent, medical and gynaecological history, vital signs, weight and height, subject satisfaction with the current method of contraception if any, overall health status through the validate questionnaire SF-12^® ^Health Survey [32,33], pregnancy test), followed by visits after cycle 1 (Visit 2), cycle 3 (Visit 3), and cycle 6 (Visit 4) of treatment were scheduled. Pregnancy test was done in V1 and repeated at the end of study (Visit 4, or early withdrawal), or earlier if pregnancy was suspected (missed withdrawal bleeding). At Visits 3 and 4, each subject was asked to complete a set of questions concerning her satisfaction with the contraceptive patch, as well as questions about her overall health status, incorporating the SF-12^® ^Health Survey. Adverse events, concomitant medications and body weight were recorded throughout the study. A further contact (telephone or visit) was also performed between Visit 1 and 2 to confirm subject's start of menses and of study medication. Due to a local amendment, at each visit, Italian Investigators assessed women's body composition with bioelectrical impedance analysis: these results have been recently published in a nutrition journal [34].

### Participants

Healthy women aged 18 to 45 years, sexually active and at risk of pregnancy, having a regular menstrual cycle occurring every 25–35 days (except for women using an implant), not pregnant, with a normal Pap smear within the previous 12 months, agreeing to use only the assigned study drug as contraception during the study (except when back-up contraception was required for sexual transmitted disease protection, detached or delayed patch changes) and having signed the Informed Consent Form were eligible to participate in the study. Participants were excluded in cases of: known history or presence of contraindications to hormonal contraceptives; recent history of alcohol or other substance abuse; oily, irritated or damaged skin at all potential sites of application; chronic use of barbiturates, anti-epileptics, rifampin, griseofulvin, or other hepatic enzyme-inducing drugs or of systemic antibiotics; concurrent use of an oestrogen and/or progestin-containing medication; any experimental drug and/or experimental device within 30 days prior to the screening visit.

### Study medication

Eligible subjects applied the first patch on the first day of menses (immediately after removal of an implant, 12 weeks-12 weeks and 5 days following the last DMPA injection) for 1 week and replaced on the same day of the week for 3 consecutive weeks. The application sites were buttocks, abdomen, upper torso or upper outer arm (excluding breasts), using each time a different place to avoid potential irritation. If the patch partly or completely detached for < 24 hours, the patch was to be re-applied to the same place or replaced with a new patch immediately without additional contraception; for > 24 hours or if the subject was not aware when the patch had lifted or become detached, the subject started a new cycle of 3 weeks, using a non-hormonal back-up method for the first 7 days.

### Study evaluation

Subject satisfaction after 3 and 6 cycles of patch use was assessed by the Measures of Satisfaction, General Health and Wellbeing questionnaire, including the SF-12^® ^Health Survey. The Investigator gave a paper questionnaire, translated in local language, to each woman and she filled in it during the visit. When applicable, a comparison was made between the patch and the method used at study entry. The subject's post-study contraceptive choice was also recorded. Compliance was assessed at all visits by inspection of returned study medication boxes and review of Diary Cards. Conflicting information between the two sources of data were analysed, discussed with the woman and then fixed by the Investigator during each visit; if necessary corrections to Diary Cards were made under the supervision of the Investigator. Adherence was calculated using the verified data from Diary Cards. A perfect compliance cycle was defined as 21 consecutive days of patch use with no patch worn longer than 7 days and the patch-free interval minimally 1 and maximally 7 days. A subject compliant score (%) was calculated as number perfect compliant cycles/number ITT cycles per each participant (ITT cycles defined as all the cycles of the ITT population, i.e. women who used at least 1 study patch during the trial, regardless of whether the patch was used at all during the cycle).

The Pearl Index (number of pregnancies per 100 woman-years of product use) and life table analysis (gross cumulative probability of pregnancy) were used to assess contraceptive efficacy. Adverse events were reported and recorded from the time of the first study-related procedure to the time of the last study-related procedure.

### Statistical analysis

In Italy about 200 subjects were planned to enter the study. As defined by the study protocol, all subjects who used at least 1 study patch were considered enrolled and included in the analyses (ITT population). Categorical data (medical and gynaecological history, primary contraceptive information, concomitant medications) were summarized by subject counts and percentages, and continuous variables (e.g., age, weight, scores, vital signs, BMI) by the means, medians, standard deviations, maxima, minima and numbers of subjects, as appropriate. The number and percentage of subjects in each age class (i.e., ≤ 21, 22–25, 26- ≤ 30, 31- ≤ 40, 41- ≤ 50, >50) were tabulated. Differences of continuous and ordinal variables (satisfaction degree, quality of life questionnaire SF-12^®^, personal preference, future contraceptive use, application site and patch removal, compliance) were analyzed within group with the Wilcoxon signed rank test, and between groups with the Wilcoxon two-sample test. Between groups differences of categorical variables were tested by means of the Fisher exact test. Statistical significance was declared at the 5% level (2-sided).

The Pearl Index (determined from the number of on-therapy pregnancies multiplied by 1300, divided by the number of cycles on therapy) was calculated for the ITT subset and for the Perfect Compliance subset of subjects. A 95% confidence interval of the Pearl index was calculated using the Poisson method. Cumulative pregnancy rates were calculated by the Kaplan-Meier method. The number and percentage of subjects with at least one adverse event were tabulated. Tables of the type and incidence of adverse events were presented using the WHO system organ class.

## Results

### Demographic and baseline characteristics

Two hundred and seventeen women were screened in Italy; 207 used at least one patch, they were considered enrolled and they were included in the analyses (ITT population). Because all subjects (207 women) provided diary data and had (minimally) 1 follow up visit, the endpoint could be calculated for all subjects. 175 women (84.5%) completed the 6-month study; of the 32 dropouts (47% of whom discontinuing after Cycle 1), 11 withdrew because of adverse events, 10 for subject's choice, 4 were lost to follow-up, 1 became pregnant and 6 for other reasons. Considering the discontinuation rate by cycle, 15 women discontinued the study after cycle 1, 8 women after cycle 2, 7 women after cycle 3 and 2 women after cycle 4. In summary, V1, V2 and diary data are available for 207 women who used at least one patch, as well as V3 data for 189 women and V4 for 177 women; finally, 175 women completed the 6-cycle study.

The subjects' baseline characteristics are summarized in Table 1. The mean age of the women was 28.4 (SD 6.4) years, divided into the following age classes: 31 to 40 years: 30.4%; 26 to 30 years: 29.5%; 22 to 25 years: 18.8%; 18 to 21 years: 16.9% and 41 to 45 years: 4.4%. About 79% of the women had a BMI class of 18.5 to 25 kg/m^2 ^and only 5 subjects had a BMI above or equal to 30 kg/m^2^. Sixty-one subjects (29.5%) smoked an average of 8.4 (SD 6.3) cigarettes per day.

**Table 1:** Baseline subjects' characteristics (N = 207)

**DEMOGRAPHIC CHARACTERISTICS**		**Mean ± SD**	
**Age, years**		28.4 ± 6.4 (range 18 – 45)	
**Weight, Kg**		57.9 ± 8.8 (range 42 – 110)	
**Height, cm**		163.2 ± 6.1 (range 147 – 180)	
**Body Mass Index, Kg/m^2^**		21.8 ± 3.1 (range 15 – 38)	
**Pulse, beats per minute**		72.4 ± 6.7 (range 54 – 91)	
**Systolic blood pressure, mmHg**		112.8 ± 9.6 (range 80 – 140)	
**Diastolic blood pressure, mmHg**		70.7 ± 7.0 (range 58 – 85)	
**Smokers (n, %)**		61 (29.5)	

**OBSTETRIC AND GYNAECOLOGICAL HISTORY**		**Mean ± SD**	

**Cycle length, days**		28.6 ± 1.7 (range 25 – 35)	
**Period length, days**		4.7 ± 1.1 (range 2 – 8)	

**Pregnancies (n, %)**	**Deliveries (n, %)**	**Women (n, %)**	**Women (n, %)**

0	0	129 (62.3)	143 (69.1)
1	1	31 (15.0)	29 (14.0)
2	2	28 (13.5)	29 (14.0)
3	3	12 (5.8)	6 (2.9)
4	4	7 (3.4)	-

**Contraceptive methods currently used at baseline (n, %)**		**Women (n, %)**	

None		125 (60.4)	
Oral contraceptive		37 (17.9)	
Vaginal ring		2 (1)	
Intrauterine method		1 (0.5)	
Withdrawal method		3 (1.4)	
Natural family planning method		1 (0.5)	
Barrier method		35 (16.9)	
Not reported		3 (1.4)	

**Degree of satisfaction with the contraceptive methods currently used at baseline (n = 82, %)**		**Women (%)**	

Very satisfied		6.1	
Satisfied		39.0	
Neither satisfied nor dissatisfied		28.0	
Dissatisfied		23.2	
Very dissatisfied		3.7	

At screening visit, 18% of the subjects reported one or more currently active concomitant morbidities, mainly involving the allergic/immunologic (10%), the gastrointestinal (3%), the endocrine (2%), the dermatological (2%) and the genito-urinary (2%) system. One hundred and twenty-nine subjects (62.3%) were nulligravida, 143 (69.1%) were nullipara (Table 1) and the mean numbers of pregnancies and deliveries were 0.7 and 0.5, respectively.

Overall, 98/207 subjects (47.3%) had used a contraceptive method in the 3 months prior to study entry: oral contraceptives (45 women, 21.7%), barrier methods (43 women, 20.8%), other methods (10 subjects, 4.8%).

At study entry, 82 women (39.6%) were still using a contraceptive method (Table 1). Using a five point scale questionnaire, these subjects expressed their opinion regarding convenience, inconsistent use, worry about getting pregnant, incorporation into their lifestyle, frequency of use, feeling secure, improvement of sex life, acceptance by their partner and overall satisfaction. Only 30.5% of women judged their method as convenient, 39% of them never forgot it and 68.3% was never or minimally worried about getting pregnant despite the method; 50 women (60.9%) agreed on the easy incorporation of the method into their lifestyle and 33 (40.2%) were satisfied with the frequency of method use. In 67.1% of cases women felt confident about the security of the method, but only in about one third of the cases the used method improved their sexual life. Overall, 45.1% of the subjects were very satisfied/satisfied, while 26.9% of them were very dissatisfied/dissatisfied with their current contraceptive method (Table 1).

### Compliance

Of the 1110 ITT cycles, 1034 (93.2%) were perfectly compliant. The mean subject compliant score was 90%. 134/175 (76.6%) subjects had 6 cycles perfectly compliant. Buttocks (50.8%) and abdomen (30.6%) were the most used application sites. One hundred and fifty-nine subjects changed the application site at least once during the study; the most common reasons for patch removal was a scheduled change (92%), partial detachment (4.2%), complete detachment (3%), skin reaction (0.3%).

### Satisfaction

**Figure 1:**
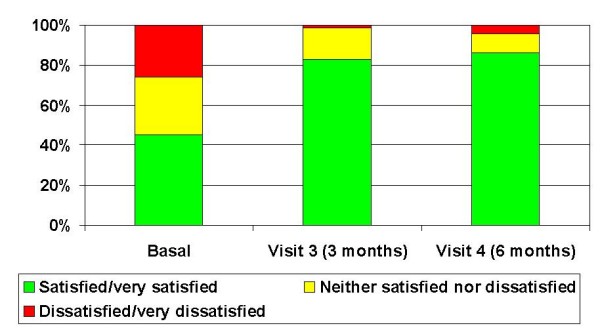
**Comparison between satisfaction with the contraceptive patch and with other methods used at baseline**.

At Visits 3 and 4, a satisfaction questionnaire was filled in: the results are summarized in Table 2. Overall, the majority of the subjects responded favourably about all the aspects. At the end of the last cycle, almost 90% considered the transdermal contraception very convenient/convenient, 96% appreciated once-a-week frequency and 95% reported they easily incorporated the patch into lifestyle. The scores at Visit 4 did not differ statistically significantly from the Visit 3 scores, except for the satisfaction with the frequency of application, which increased from Visit 3 to Visit 4 (p < 0.01). Concerning satisfaction versus the baseline method, the rate of subjects very satisfied or satisfied with their contraceptive method increased from 45.1% at baseline to 82.9% at Visit 3 and 86.3% at Visit 4 (Figure 1).

**Table 2:** Subjects' responses to questions about satisfaction with the contraceptive patch

**Response**	**Visit 3** **(3 months)** N (%)^a^	**Visit 4** **(6 months)** N (%)^b^
Patch was convenient/very convenient	163 (81%)	155 (88%)
Worried a little/none of the time about getting pregnant	173 (86%)	155 (88%)
Satisfied/very satisfied about adhesion of patch	159 (79%)	150 (85%)
Satisfied/very satisfied about adhesion of patch in heat/humidity	138 (68%)	127 (72%)
Satisfied/very satisfied with choice of 4 application sites	161 (80%)	148 (84%)
Agreed/strongly agreed easily incorporated patch into lifestyle	177 (88%)	169 (95%)
Agreed/strongly agreed satisfied with once-a-week frequency	184 (91%)*	169 (96%)*
Agreed/strongly agreed felt secure that patch works	165 (82%)	158 (89%)
Agreed/strongly agreed patch improved sex life	101 (50%)	102 (58%)
Agreed/strongly agreed partner accepted patch	152 (75%)	140 (79%)
**Overall satisfaction with the patch**	**167 (83%)**	**156 (88%)**

a On a total of 202 subjects

b On a total of 177 subjects

*P < 0.01 V4 vs V3

### Preference, Quality of Life and future contraception

**Figure 2:**
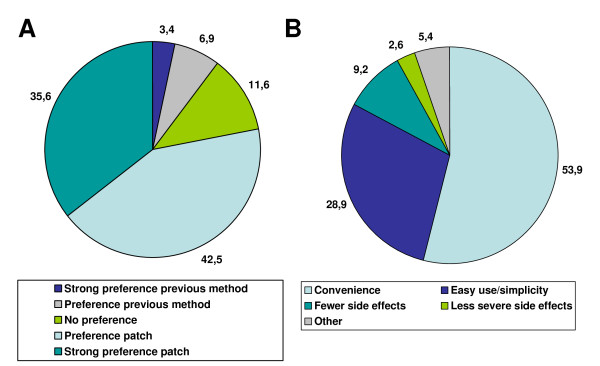
**Treatment preference at study end (A) and reasons for contraceptive patch preference (B)**.

Comparing the transdermal patch with the primary contraceptive method used in the 3 months before, 68/87 subjects (78.1%) had a preference or strong preference for the patch (Figure 2); the main reasons for this choice were convenience (53.9%), ease of use/simplicity (28.9%) and fewer or less severe side effects (11.8%). 30 subjects (75%) who used oral contraceptives and 34 subjects (87.2%) previously using barrier methods as their primary method of contraception in the previous 3 months, had a preference or strong preference for the patch. Overall, the percentage of subjects with preference for the patch was greater for subjects that completed the study (66/81, 81.5%) than for those who did not, though information about their preference was available only for 6 non-completers, 2 of them had a preference for the patch.

The indication of preference for the transdermal patch was consistent with the satisfaction score.

Quality of Life Questionnaire SF-12 included Physical Component Summary (PCS) and Mental Component Summary (MCS) evaluation. The mean PCS value was stable during the study visits, although the mean MCS was statistically higher at Visits 3 and 4, and at endpoint than at baseline (p < 0.001, p = 0.0049, and p < 0.05, respectively).

When questioned about their future contraceptive use, 114 out of 201 subjects (56.7%) specified that they would use the transdermal patch, 46 (22.9%) did not intend to use any contraceptive method and only 19 subjects (9.5%) would prefer to use an oral contraceptive. Almost all subjects who would use the patch as their future contraceptive method completed the study (111/114; 97%).

### Efficacy

Contraceptive efficacy was assessed for all the participants who used at least 1 study patch. One on-therapy pregnancy occurred during the study, presented as a pregnancy due to "method failure" because, from the subject's Diary Cards, there was no 100% certainty that the patch was replaced more than 24 hours after a detachment (perfect compliance demanded that a detached patch should be replaced within 24 hours; just in case of proved inconsistent use a pregnancy can be considered "user failure"). Globally, the Pearl Index for the ITT population, based on 1110 cycles, was 1.17, 95% CI (-1.12, 3.47); the Pearl Index for the Perfect Compliance subset, based on 1034 cycles, was 1.26, 95% CI (-1.21, 3.72). Kaplan Meier estimates of the cumulative probabilities of pregnancy were determined to be 0.57% for the ITT subset and 0.60% for the Perfect Compliance subset, respectively.

### Safety

Overall, the subject's mean pulse, systolic and diastolic blood pressure did not change significantly during the study. The mean increase in body weight from baseline to Visit 4 was minimal (0.7 kg), while the mean BMI increased by 0.3 kg/m^2^. These increases were statistically significant (p < 0.0001), but were not considered clinically relevant.

Seventy-one subjects (34.3%) experienced one or more adverse events during the 24 weeks of treatment. The most frequently reported adverse events are summarized in Table 3. About 93% of adverse events were mild (63.9%) or moderate (29.2%) in severity, with only 6.9% judged as severe. In 28.5% of cases no correlation to the study medication was found and only 13.2% of the events were considered very likely related to the patch. No serious adverse events or deaths occurred during the study period.

**Table 3:** Most commonly adverse events occurred during the study period (> 2% of the subjects)

**Adverse events**	**N**	**% of women**
Headache	21	10.1
Spotting between menses	9	4.3
Itching	7	3.4
Nausea	6	2.9
Breast pain	6	2.9
Localised skin reaction	6	2.9
Breast tension	5	2.4

Overall, only 11 women (5,3%) withdrew from the study because of adverse events due to localized skin reactions (3 cases), headache (2 cases, one of which associated with vomiting), allergy, anxiety with nausea and rash, breast pain, itching, decreased libido and spotting between menses (1 case each).

**Figure 3:**
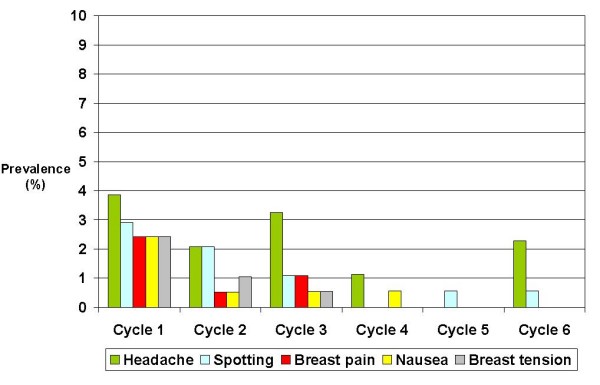
**Prevalence in each cycle of the most frequently mentioned adverse events (> 2% of the subjects)**.

The frequency of adverse events was higher during the first cycle: 49.3% of adverse events started during the first cycle of the study, but 34% of them stopped during the same cycle. It is noteworthy that the frequency decreased over time; in fact, only 20% of emergent adverse events started after third cycle. The most prevalent adverse events in cycle 1 were headache (4%) and spotting between menses (3%), and by cycle 4 their prevalence decreased to 1% and 0%, respectively (Figure 3).

## Discussion

The primary objective of this multicentre study was to evaluate satisfaction, preference, compliance and well-being following use of the contraceptive patch, in a naturalistic setting as close to the real-life situation as possible ("real world" use), in order to have data reflecting how the patch is normally used by European/Italian women.

In Italy, after 6 cycles of patch treatment, the satisfaction rate in all women was 88%; considering only the women using a contraceptive method at baseline, their satisfaction increased from 45% with the previous method to 86,3% with the patch while the dissatisfaction decreased, respectively, from 27% to 4%. This is in agreement with other studies with the contraceptive patch: Wan reported significantly higher levels of satisfaction in the women who used the contraceptive patch compared to the oral contraceptive users (OR = 2.05; p = 0.001) [35], while Weisberg showed that out of 279 participants who completed 9 cycles, 91% were satisfied with the patch [36]. In an international randomized trial conducted on 1489 women, significantly more users were very satisfied with the contraceptive patch compared to oral contraceptives (p > 0.05) and, in contrast to oral contraceptives, the percentage of women being very satisfied with the patch, gradually increased with age, resulting in a more pronounced difference in women aged > 34 [37]. Finally, 97% of adolescents evaluated in a recent study were very satisfied/somewhat satisfied with the contraceptive patch and 93% of them stated that they would recommend it to a friend/relative [22].

Concerning the comparison with the method used in the 3 months before study enrolment, 78.1% of subjects had a preference/strong preference for the patch due to convenience, ease of use/simplicity and fewer side effects of the patch over the other methods; similarly, Weisberg reported that almost 3 out of 4 participants preferred the patch to their previous method [36]. In this regard, 93% of adolescents reported that they remembered to apply the patches on time, and 40% of them stated that its use was easier than that of previous contraceptive methods [22].

The high level of satisfaction and preference with transdermal contraception may be considered important when trying to maximize long-term compliance. In this study, compliance was very high (93,2% perfectly dosing cycles), in accordance with the pooled analysis of randomized controlled trials (88.7% vs 79.2% of oral contraceptives, p < 0.001). In the observational study conducted in Canada, patch perfect compliance was high across all cycles (88%) and did not differ significantly across age [36]. Similarly, Urdl demonstrated that compliance was consistently better in the patch group (range 90 to 97%) compared to the oral contraceptive group (85 to 92%) [37]. The once-weekly application schedule for the patch is desirable because it is well suited to the lifestyle of European women, including younger women for whom compliance with oral contraceptives is a particular issue [20,37].

The improved compliance observed with the patch may lead to fewer unintended pregnancies [8,16,21], although confirmation of this would require additional clinical studies. Only 1 pregnancy was reported and occurred during the study (confirmed by urine and blood positive pregnancy tests, gynaecological examination and ultrasound). These data are consistent either with those of the pooled analysis [18] or with the more recent multicentre studies, evaluating a high number of women in conditions very close to real use in daily life [36,37].

In this study, the reporting frequency for each adverse event was lower than rates for the same adverse events reported in other clinical trials. Headache was the most frequently reported event, followed by spotting between menses, itching, breast pain, localized skin reaction, nausea, and breast tension. In our study, application-site reactions (2.9%) were significantly lower than the 17.4% in the phase III studies pooled analysis [24] and the 13.8% reported by Urdl [37]. According to Weisberg, application site reaction was the most common patch-related adverse event in cycle 1, but diminished greatly in the successive cycles [36]. In general, < 2% of participants discontinued patch treatment for this reason [24,37].

Severity of the events, withdrawal due to safety and improvement of tolerability with progressive cycles were consistent with, or better than, those seen in previous studies [24,36,37]. In aggregate, these safety data indicate that the patch is well tolerated, with an adverse event profile similar to oral contraceptives [24,37].

The main strength of our findings is the fact that this is the first multicentre Italian study specifically designed to evaluate women's satisfaction and preference with the contraceptive patch in comparison to the previous methods. Even if several trials evaluated these items in Europe (but not in Italy) and in South Africa [37], in the United States [35] and in Canada [36], our data are the first published regarding Italian women. The open study design with broad selection criteria enabled data to be gathered on the patch in everyday woman use, with the minimum of interventions, and the results reflected how the contraceptive patch is normally used in Italy. These results can be considered innovative if we take into account that the choice and the preference for a contraceptive method could be different from country to country and influenced by different factors such as cultural and religious ones.

However, our study has also several limitations. Firstly, this is a non-randomized observational trial. This kind of approach can introduce bias, because a subject could be unconsciously predisposed to favour the new therapeutic option. But, in this study, only 50% of participants adopted a contraceptive method in the 3 months before enrolment, and the degree of satisfaction at study end was very high in all the subjects. In addition, an open-label, single arm design was chosen to reflect how women under "real world" circumstances normally use the contraceptive patch. Secondly, the characteristics of women who perfectly adhere to the regimen, as well the possible factors associated with the women who were very satisfied/satisfied with the patch, were not investigated in details; however, considering the relatively small number of our population (207 women, 175 of them are completers), we think that this type of analysis (e.g. analysis per age classes, per number of previous pregnancies/deliveries) could not be feasible and/or significant from a statistical point of view. Thirdly, women could use condoms during the study and a monogamous relationship without the need for STI protection was not an eligibility requirement. This strict lifestyle criterion was not adopted because the objective of this study was to evaluate the real-life use of the patch and not to collect information on a restricted population of women (observational study, without any registration purpose). For this reason, information about condom use, as well as the number of intercourses by cycle, were not collected. Finally, because all of the women agreed to use the patch, the adherence and acceptability results might not be representative of the general population, with a possible over-estimation of the satisfaction and adherence. By contrast, also in this case, the study reflects real-life situation: in clinical practice, woman are usually used to discussing the choice of a contraceptive method with their own Gynaecologist/GP, and this mutual decision making with her doctor is very important to reach a "therapeutic alliance", valuable predictor of patient's adherence and persistence.

## Conclusion

In addition to clinical efficacy in preventing unintended pregnancies, the choice of a contraceptive method should take into account other important attributes of the treatment that impact on compliance, such as women's preference, satisfaction, well-being and ease of use. Specifically, satisfaction with the contraceptive method is essential for long-term adherence because human behaviour tends to be repeated when it is rewarding (e.g. when the method satisfies the personal needs of the woman and her partner). In this sense, transdermal contraception has provided an important new option for many women. The EE/NGMN contraceptive patch is as effective and well tolerated as oral contraceptives; the convenience and simplicity of a weekly change schedule for the patch may contribute to its high degree of satisfaction. In conclusion, high satisfaction levels, coupled with excellent compliance, make the contraceptive patch a reasonable option for women desiring hormonal contraception.

## Competing interests

This study was sponsored by Janssen-Cilag EMEA and funded by Janssen-Cilag Italy. Silvia Marelli and Davide Sonnino belong to the Medical Affairs Department of Janssen-Cilag Italy. The other authors declare that they have no competing interests.

## Authors' contributions

PGC made substantial contributions to conception and design of the study, he was the Principal Investigator of the Italian coordinating site and contributed to data interpretation and critical revision of the manuscript; CN, SR and VB were the Principal Investigators of three Italian sites, they gave important contributions to acquisition/interpretation of data and manuscript revision; SM and DS were the Local Trial Coordinators of the study in Italy, they were involved in data interpretation and in drafting/revising the manuscript critically for important intellectual content. All authors have read and approved the final manuscript.

## Pre-publication history

The pre-publication history for this paper can be accessed here:


